# Automated Water Quality Survey and Evaluation Using an IoT Platform with Mobile Sensor Nodes

**DOI:** 10.3390/s17081735

**Published:** 2017-07-28

**Authors:** Teng Li, Min Xia, Jiahong Chen, Yuanjie Zhao, Clarence de Silva

**Affiliations:** 1 Department of Mechanical Engineering, University of British Columbia, Vancouver, BC V6T 1Z4, Canada; minxia@mech.ubc.ca (M.X.); jhchen@mech.ubc.ca (J.C.); desilva@mech.ubc.ca (C.d.S.); 2 Faculty of Science, University of British Columbia, Vancouver, BC V6T 1Z4, Canada; zhao.yuanjie@yahoo.com

**Keywords:** water quality monitoring, IoT platform, survey planner, quality indexing

## Abstract

An Internet of Things (IoT) platform with capabilities of sensing, data processing, and wireless communication has been deployed to support remote aquatic environmental monitoring. In this paper, the design and development of an IoT platform with multiple Mobile Sensor Nodes (MSN) for the spatiotemporal quality evaluation of surface water is presented. A survey planner is proposed to distribute the Sampling Locations of Interest (SLoIs) over the study area and generate paths for MSNs to visit the SLoIs, given the limited energy and time budgets. The SLoIs are chosen based on a cellular decomposition that is composed of uniform hexagonal cells. They are visited by the MSNs along a path ring generated by a planning approach that uses a spanning tree. For quality evaluation, an Online Water Quality Index (OLWQI) is developed to interpret the large quantities of online measurements. The index formulations are modified by a state-of-the-art index, the CCME WQI, which has been developed by the Canadian Council of Ministers of Environment (CCME) for off-line indexing. The proposed index has demonstrated effective and reliable performance in online indexing a large volume of measurements of water quality parameters. The IoT platform is deployed in the field, and its performance is demonstrated and discussed in this paper.

## 1. Introduction

Monitoring programs of aquatic environments play a critical role in various water uses, such as the study of aquatic life, livestock watering, human usage, irrigation, recreation, and so on. Clean water sources are beneficial not only for the aquatic ecosystem and natural habitats, but also for public health. In the past, water quality evaluation has relied primarily on time-consuming and human-intensive field measurements for data collection. Technicians usually test water sources in the field utilizing hand-held devices, or transport water samples to laboratories for further analysis. The monitoring programs of this type have been limited by their inadequate measurements on both temporal and spatial scales.

Recent advances in the technologies of sensing, robotics, and Internet of Things (IoT) have led to significant progress in the applications of environmental telemonitoring. In the field of aquatic monitoring, static stations or buoys with capabilities of automated measuring, data logging and wireless transmission have been widely designed by research institutes [1,2] or deployed by environmental departments [3,4]. Although online data gathering can be achieved by utilizing these systems, they have been limited by their inadequacy and inflexibility in spatiotemporal quality evaluation. In the past decade, sensor nodes that can carry out mobile sensing have been investigated to facilitate flexibility for gathering information at locations of interest over a large-scale area. Monitoring systems with Mobile Sensor Nodes (MSN), Unmanned Surface Vehicles (USV), or Autonomous Underwater Vehicles (AUV) have been developed and deployed to provide spatiotemporal measurements of water sources such as pools, lakes, reservoirs, rivers, and oceans. The main of research in this area has focused on system design and development [5,6,7,8], sensor deployment and path planning [9,10,11], environment modeling and field reconstruction [12,13,14,15], data interpretation and evaluation [16,17], and so on. 

This paper presents the design and development of a rapidly deployable IoT platform for the telemonitoring of surface water with regard to quality characteristics. The rapid deployment framework has to be fast and easy to deploy and maintain [18]. It may be deployed in the field only for a relatively short-term, but can achieve high-resolution spatiotemporal sampling. The data collected in this approach can benefit aquatic environmental monitoring in various applications such as the survey of an unknown area for collecting useful knowledge to establish an environmental model, the design of a sensor deployment strategy for long-duration monitoring, an analysis of microaquatic environmental changes, and so on. In the present work, an efficient survey planner and an effective online water quality index are proposed and integrated into the developed platform.

To interpret the quality profile of the study area in terms of multiple water quality parameters, the selection of the data Sampling Locations of Interest (SLoIs) is key for carrying out an automated measuring process across the study area. The distribution of the surveyed SLoIs requires a sampling frame that allows for the generation of a reliable interpretation of attributes of interest in a study area [19]. The SLoIs are generally distributed evenly over the study area or selected at some target locations that are generated based on prior knowledge of the study environment, i.e., environmental models or data-driven rules. After obtaining the objective sampling locations, paths have to be determined for the mobile sensor nodes to visit them. In the past, approaches of the Travelling Salesman Problem (TSP) have been widely applied for planning the paths to visit the objective sampling locations [20,21,22]. In the present work, the primary goal of the proposed planner is the selection of the SLoIs and the generation of a path to visit them. Specifically, a hexagonal grid-based survey planner is proposed. Given energy and time budgets, an effective and efficient path is generated to visit the SLoIs, which are uniformly distributed over the study area. This path is formed as a ring by a Minimum Spanning Tree (MST)-based path planning approach under the hexagonal tessellation. Then sub-paths extracted from the path ring are assigned to multiple MSNs to satisfy the time interval requirement for measuring at a SLoI.

The quality of water is evaluated based on its physical, chemical and biological parameters. With the objective of providing an overall representation of the water quality based on all measurements, effort has gone into developing Water Quality Indices (WQIs) [23]. A WQI provides a convenient way to represent the water quality by aggregating the measured data of water quality parameters into a numerical score. Then, the score is classed into a clear quality category for reporting to the technicians, managers, policy-makers, and other users. An Online Water Quality Index (OLWQI) is proposed in this paper to represent a large amount of online data for water quality indexing. It is modified by the index formulation of the state-of-the-art index, the CCME WQI, which has been developed by the Canadian Council of Ministers of Environment (CCME) [24] for off-line indexing. The CCME WQI has been widely used in water quality monitoring programs by many agencies and institutes throughout the world [25,26,27,28]. It is generally applied off-line, using data collected at low sampling rates (in the scale of month to quarter). Although the CCME WQI has been used as a possible index for data collected through automated sampling [29], according to our experiments, disadvantages exist when directly implementing it for online quality indexing. In the present work, the proposed OLWQI provides effective indexing results with a reliable sensitivity factor for large quantities of online data collected through automated sampling. The index formulations of the OLWQI are expressed in analytical form to facilitate the automatic execution on devices.

The proposed survey planner and the OLWQI have been implemented on the developed ICT platform, which consists of a group of mobile sensor nodes, a base station located on shore, and a remote server. Spatiotemporal measurements and the online quality index are provided as the monitoring results, which will be utilized for further decision-making, policy-making, and water management. To demonstrate its performance, the platform has been deployed at the Yosef Wosk Reflecting Pool in the University of British Columbia, Canada. The experimental results and the system performance are presented and discussed in this paper. The rest of the paper is organized as follows. In Section 2, an overview of the platform is presented. Section 3 presents the survey planner in the IoT platform. The proposed OLWQI is derived in Section 4. In Section 5, the hardware components of the platform are described in detail. Section 6 presents the system implementation, and demonstrates and discusses the experimental results. The final section concludes the paper.

## 2. Platform Overview

In the past decade, many IoT platforms have been developed and implemented for aquatic environmental monitoring. Concerning the number of sensing units in a system, the platforms can be categorized into two major types: systems with a single monitoring station (e.g., [3,7,8,10,11,13]) and systems with multiple sensor nodes in a monitoring network (e.g., [1,4,5,6,14,16]). A single monitoring station commonly has sufficient computation and communication resources, and a power supply. The main shortcoming of deploying a single station is its lack of ability to provide high-resolution spatiotemporal monitoring over a large geographical area. A monitoring network with multiple sensor nodes, in contrast, facilitates the monitoring process on both spatial and temporal scales. Concerning the mobility of the sensing unit in a system, the platforms can be classified into static systems (e.g., [1,3,4,5,6]) and mobile systems (e.g., [5,6,7,8,10,11,13,14]). The static platforms have sensing units deployed at predetermined locations, and provide continuous online measurements in the field. These platforms have proven to be effective in supporting the environmental monitoring in a timely manner due to their abilities regarding data requisition, information processes and wireless transmission [2]. However, they have been rather hindered by their inadequacy and inflexibility on spatial-scale sensing for area surveillance. In contrast, the mobile platforms that consist of mobile sensing units are able to operate measuring processes by travelling over a large spatial scale. These platforms provide the capability of information gathering at locations of interest over the study area. However, each mobile sensing unit often has crucial resource constraints, such as storage energy, that limit the range it can explore (spatial point of view) or the number of sampling locations it can measure before the phenomena in the monitored field varies significantly (temporal point of view) [9].

In this paper, the development of a rapidly deployable and easily maintainable IoT platform is presented. The objective of this platform is to provide effective and efficient quality evaluation of surface water in a high-resolution spatiotemporal manner. Compared to the state-of-the-art systems, the platform introduced in this paper has improved behavioral performance on several aspects. Firstly, the low cost of the components in the platform bring a cost effective solution for automated water quality evaluation. In addition, our platform achieves fast deployment and easy maintenance, which simplifies the initial deployment and follow-up maintenance procedures. Secondly, multiple MSNs facilitate the area surveillance on the spatiotemporal scale compared to the static sensing stations. More importantly, the proposed planning algorithm provides an efficient survey planner by considering the energy and time constraints. Thirdly, online quality indexing is implemented in the platform by integrating online data of multiple parameters to give a comprehensive quality evaluation of a study area.

**Figure 1:**
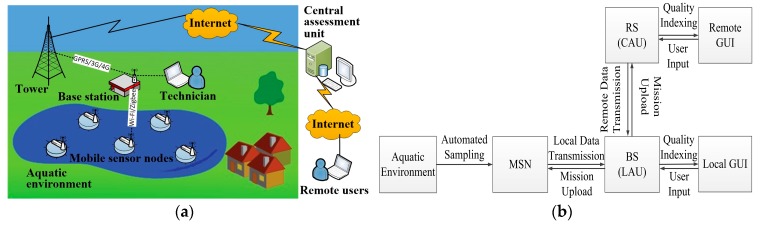
The proposed Internet of Things (IoT) platform: (**a**) Architecture of the platform for water quality monitoring; (**b**) Workflow diagram of the platform.

In the implementation, Mobile Sensor Nodes (MSNs) are deployed in a distributed way in the monitored field. The survey missions (sensing locations and moving paths) for the MSNs are generated at a Remote Server (RS) and transmitted to the MSNs via a Base Station (BS). Then, the MSNs follow the received missions to collect data at the scheduled sampling locations. Each MSN consists of a set of heterogeneous sensors to measure different water quality parameters. The collected data is then transmitted to the base station (BS) through a local wireless network (e.g., Wi-Fi or Zigbee). The monitoring results are presented at the BS via a Local Assessment Unit (LAU) in two forms: (1) the measurements in terms of water quality parameters at the sampling locations; and (2) the online water quality index. The former form presents the quantitative measurements in the field. The latter form presents the qualitative evaluation of the surface water. The results are also transmitted to the RS with a Central Assessment Unit (CAU) running on it. Thus, the monitoring results can be accessed locally at the BS by the technicians in the field or accessed remotely by the users via the Internet. The architecture and the workflow diagram of the proposed IoT platform are presented in Figure 1.

## 3. Hexagonal Grid-Based Survey Planner

The monitored surface water is generally treated as a continuous planar area. The estimation of the aquatic environmental characteristics is interpreted based on the distribution of the sampling locations across the study area. To characterize the whole study area, especially for an unknown area without any prior knowledge, data samples are generally distributed in a uniform manner. Accordingly, in many applications, a sampling frame is generally designed by decomposing the sensing domain into a grid of cells, to distribute uniform plots across the area of interest.

### 3.1. Sampling Location of Interest (SLoI)

In the present work, the sampling locations are generated by utilizing a hexagonal cell decomposition approach to distribute the plots evenly across the monitored area A. This sampling framework introduces spatially balanced sampling locations where the distances between any neighboring SLoIs are equal. Let the set $S=\left\{s_{1},\ldots ,s_{m}\right\}$ represent m SLoIs to be measured for data collection. All sample locations in S are generated using a hexagonal grid-based decomposition of a continuous planar area A with its known contour $\hat{A}$. Let the set $U=\left\{u_{1},\ldots ,u_{n}\right\}$ represent n MSNs that are deployed in a distributed manner in the monitored field. Each MSN consists of a variety of l heterogeneous sensors that measure multiple parameters.

**Figure 2:**
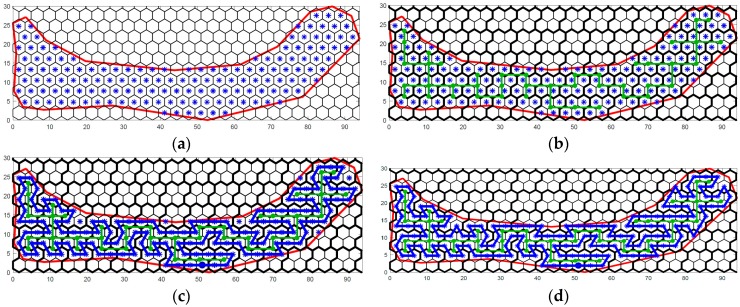
An execution example of the proposed hexagonal grid-based survey planner: (**a**) Hexagonal tessellation with Sampling Locations of Interest (SLoIs); (**b**) The created Minimum Spanning Tree (MST) vertices and the constructed relative MST; (**c**) Generation of the circumnavigation path based on the MST. The solid circle on the path denotes a selected starting point; (**d**) The overall path ring after path update.

First, the study area A is decomposed into a grid of cells by a hexagonal tessellation. The center of each hexagonal cell is chosen as a SLoI s∈S if it is located within the contour $\hat{A}$ of the study area. An example is shown in Figure 2a, where the thick red line denotes the contour $\hat{A}$, and the blue asterisks denote the SLoIs. Then, the sampling locations are created uniformly across the study area, spaced at d, with $d=\sqrt{3}r$, where d is the distance between two neighboring SLoIs, indicating the sampling resolution of the survey, and r is the edge length of a hexagonal cell. After obtaining the locations for sampling, a path is required to visit these target locations. In this work, a spanning tree-based path planning algorithm is used to travel through the SLoIs in an effective and efficient way.

### 3.2. Spanning Tree-Based Path Planning

The SLoIs are generated following the sampling frame introduced above. To measure these points, in this work, a spanning tree-based path planning approach is proposed for sensor scheduling and path planning. This approach has been originally implemented on a coverage path planning problem using square cellular decomposition [30,31,32]. In this paper, a novel spanning tree-based survey planner is proposed with hexagonal cellular decomposition. First, a Minimum Spanning Tree (MST) is constructed $T_{min}=\left(V_{T},E_{T}\right)$, where $V_{T}$ and $E_{T}$ are the sets of vertices and edges of the tree, respectively. Then, a path to visit all sampling locations is generated based on the obtained MST. 

The vertices $v_{T}\in V_{T}$ for constructing the MST are created based on a set of coarse cells. Each coarse cell contains four regular hexagonal cells. An example with a tessellation of coarse cells is shown in Figure 2b, where the polygons with thick black edges denote the coarse cells. A vertex is created based on the number and the positions of the SLoIs within a coarse cell. The bottom left, bottom right, top left, and top right regular hexagonal cells (fine cells) within a coarse cell are labeled as fine-cell 1, 2, 3, and 4, respectively. The vertex creation strategies are summarized in Table 1. For a coarse cell with only one sampling location inside, no vertex is created. After creating the vertices $v_{T}\in V_{T}$ by traversing all the coarse cells, an MST $T_{min}=\left(V_{T},E_{T}\right)$ is constructed based on these vertices. Prime’s or Kruskal’s algorithm is generally used to span a Minimum Spanning Tree. An example of the constructed MST using Kruskal’s spanning tree algorithm based on the created vertices is shown in Figure 2b. The green solid circles denote the created MST vertices, and the green lines between the MST vertices are the constructed MST edges. 

**Figure 3:**
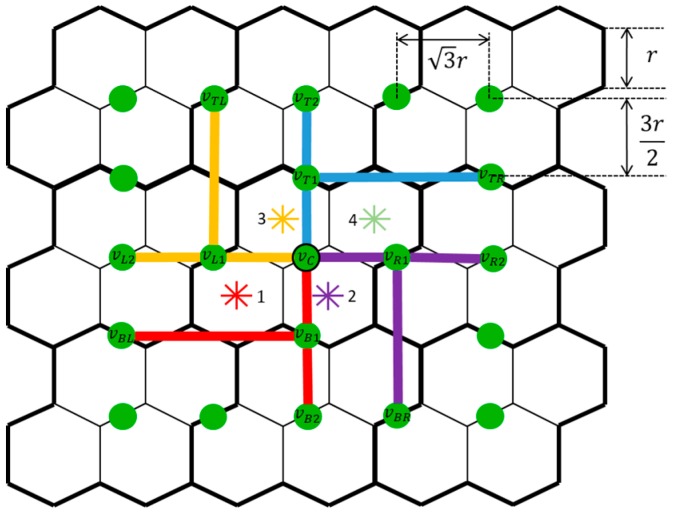
Possible MST vertices and edges surrounding a coarse cell.

A path $p=\left(sp_{1},sp_{2},\ldots ,sp_{w}\right)$ is then generated to visit sampling locations s∈S based on the constructed spanning tree $T_{min}$, where sp∈S denotes a sampling location that is visited on the path. |p| denotes the total length of the path. A MSN travels through the SLoIs following the sequence order $\left(sp_{1},sp_{2}\ldots ,sp_{w}\right)$. $SP=\left\{sp_{1},sp_{2},\ldots ,sp_{w}\right\}$ represents the set of visited SLoIs and |SP| denotes the number of the sampling locations in the set. The path starts at a predetermined starting location $sp_{1}$, then visits the SLoIs by circumnavigating the constructed MST clockwise or counter-clockwise, and finally returns to the starting location, forming a path ring. Starting from $sp_{1}$, the planner iteratively finds the next target location (the next neighboring location to visit) according to the current location and generates a path segment between the current location and the target location until it reaches $sp_{1}$ again. In each iteration, the fine-cell position of the current location inside its coarse cell is first identified. One of the four possible positions (fine-cell 1, 2, 3, or 4 in its coarse cell, see the asterisks in Figure 2) can be identified for a given current location. The rules to find the next target location are based on the conditions of its surrounding MST vertices or edges. In Figure 3, the green solid circles and lines denote the possible MST vertices and tree edges surrounding the central coarse cell with respect to the four possible fine-cell positions. The proposed algorithm for clockwise circumnavigation is given in Algorithm 1 with pseudo code.

**Algorithm 1:** clockwiseCircumnavigationPathGeneration**Input**: $S,T_{min}=\left(V_{T},E_{T}\right),sp_{1}\in S$ **Output**: $p=\left(sp_{1},\ldots ,sp_{w}\right),sp\in S$ 1  $v_{current}=sp_{1}$; 2  **do** 3    $v_{C}=getCoarseCellLocation\left(v_{current}\right)$; 4    **switch**
$getCellPosition\left(v_{current}\right)$
**do** 5      **case** 1 **do** 6        **if**
$e_{v_{B1},v_{BL}}\in E_{T}$
**then**
$v_{next}=moveTo\left(\prime \mathrm{Left}\prime \right)$; 7        **else if**
$e_{v_{C},v_{B1}}\in E_{T}\left|\left|e_{v_{C},v_{B2}}\in E_{T}\right|\right|e_{v_{B1},v_{B2}}\in E_{T}$
**then**
$v_{next}=moveTo\left(\prime \mathrm{Bottom}\;\mathrm{Right}\prime \right)$; 8          **else if**
$v_{C}\in V_{T}\;$**then**
$v_{next}=moveTo\left(\prime \mathrm{Right}\prime \right)$; 9            **else**
$v_{next}=moveTo\left(\prime \mathrm{Top}\;\mathrm{Right}\prime \right)$; 10     **case** 2 **do** 11       **if**
$e_{v_{R1},v_{BR}}\in E_{T}$
**then**
$v_{next}=moveTo\left(\prime \mathrm{Bottom}\;\mathrm{Right}\prime \right)$; 12       **else if**
$e_{v_{C},v_{R1}}\in E_{T}\left|\left|e_{v_{C},v_{R2}}\in E_{T}\right|\right|e_{v_{R1},v_{R2}}\in E_{T}$
**then**
$v_{next}=moveTo\left(\prime \mathrm{Right}\prime \right)$; 13         **else if**
$v_{C}\in V_{T}\;$**then**
$v_{next}=moveTo\left(\prime \mathrm{Top}\;\mathrm{Right}\prime \right)$; 14           **else**
$v_{next}=moveTo\left(\prime \mathrm{Left}\prime \right)$; 15     **case** 3 **do** 16       **if**
$e_{v_{L1},v_{TL}}\in E_{T}$
**then**
$v_{next}=moveTo\left(\prime \mathrm{Top}\;\mathrm{Left}\prime \right)$; 17       **else if**
$e_{v_{C},v_{L1}}\in E_{T}\left|\left|e_{v_{C},v_{L2}}\in E_{T}\right|\right|e_{v_{L1},v_{L2}}\in E_{T}$
**then**
$v_{next}=moveTo\left(\prime \mathrm{Left}\prime \right)$; 18         **else if**
$v_{C}\in V_{T}\;$**then**
$v_{next}=moveTo\left(\prime \mathrm{Bottom}\;\mathrm{Left}\prime \right)$; 19           **else**
$v_{next}=moveTo\left(\prime \mathrm{Right}\prime \right)$; 20     **case** 4 **do** 21       **if**
$e_{v_{T1},v_{TR}}\in E_{T}$
**then**
$v_{next}=moveTo\left(\prime \mathrm{Right}\prime \right)$; 22       **else if**
$e_{v_{C},v_{T1}}\in E_{T}\left|\left|e_{v_{C},v_{T2}}\in E_{T}\right|\right|e_{v_{T1},v_{T2}}\in E_{T}$
**then**
$v_{next}=moveTo\left(\prime \mathrm{Top}\;\mathrm{Left}\prime \right)$; 23         **else if**
$v_{C}\in V_{T}\;$**then**
$v_{next}=moveTo\left(\prime \mathrm{Left}\prime \right)$; 24           **else**
$v_{next}=moveTo\left(\prime \mathrm{Bottom}\;\mathrm{Left}\prime \right)$; 25   $v_{current}=v_{next}$; 26 **while**
$v_{current}=sp_{1}$;

An execution example of the Algorithm 1 is shown in Figure 1c. A path ring (the thick blue lines between SLoIs) is generated to circumnavigate the MST and visit the SLoIs inside the fine-cells. In this figure, some SLoIs are not visited by the generated circumnavigation path in Algorithm 1. To cover the remaining SLoIs, a simple strategy is proposed. For an unvisited SLoI, if there is a path segment between its two neighboring locations (these two locations are also neighbors), first generate two path segments between the current location and those two locations, and then remove the path segment between those two locations. By applying this strategy, the updated overall path ring is shown in Figure 1d.

Given a hexagonal cell size r, $d=\sqrt{3}r$, the total number and locations of the SLoIs are determined according to the sampling frame introduced in subsection A. There is an upper bound for the total length of the generated path |p|: |p|≤num·d, where num=N(r) represents the number of SLoIs inside the study area with respect to the cell size r. Since the energy budget $e_{bdt}$ indicates the total length that an MSN can travel, for simplicity, the present paper utilizes the energy budget $e_{bdt}$ with a length unit. Accordingly, given the area contour $\hat{A}$ and the energy budget $e_{bdt}$, a feasible cell size r can be determined such that $num\cdot \sqrt{3}r\leq e_{bdt}$. Then the proposed planner will generate a path under the sampling density d, satisfying the energy constraint $\left|p\right|\leq e_{bdt}$. However, for an arbitrary area contour $\hat{A}$ there is no analytic expression num=N(r) with respect to a cell size r. Thus, it is difficult to find the maximum sampling density such that $num\cdot \sqrt{3}r\leq e_{bdt}$. In an application, cell size r can be obtained by iteratively decreasing from an initial value $r_{0}$ to find a feasible r close to its minimum value such that $num\cdot \sqrt{3}r\leq e_{bdt}$.

At a sampling location s∈S, data collected by an MSN is expressed as $x_{s}=\left[x_{1}^{s},x_{2}^{s},\ldots ,x_{l}^{s}\right]$, where x denotes the measurements of the l parameters. It is clear that the data is collected at different locations sp∈S along the planned path p one by one in a time series. Let $t_{sp_{i},sp_{j}}$ represent the time consumption when traveling from sampling location $sp_{i}$ to $sp_{j}$. In order to avoid the water current disturbances induced by moving, a MSN stops and stays at a SLoI while carrying out the measurement process. Let $t_{M}$ represent the time taken by the measurement process for staying at an SLoI. The period that all SLoIs are visited once by a MSN is called a survey cycle or a sampling cycle. If a single MSN is deployed to follow the generated path ring $p=\left(sp_{1},\ldots ,sp_{w}\right)$ periodically, it leads to a time consumption $t_{p}=\left|SP\right|\cdot t_{M}+\sum_{i=1}^{w-1}t_{sp_{i},sp_{i+1}}$ for each sampling cycle. It means each SLoI is visited at every time interval $t_{p}$. To ensure the sampling rate at each SLoI satisfies a given time interval (time budget) $t_{bdt}$, if $t_{p}>t_{bdt}$, the path ring p is divided into $n\in Z^{+}$ sub-paths, such that

(1) $$\frac{\left|SP\right|\cdot t_{M}}{n}+\frac{\left|p\right|}{n\cdot v}\leq t_{bdt}.$$

where v is the average speed of a MSN. To find the minimum number of MSNs to be deployed, $n=\lceil \left(\left|SP\right|\cdot t_{M}\right)/t_{bdt}+\left|p\right|/\left(v\cdot t_{bdt}\right)\rceil$ is obtained. The path ring p is uniformly divided into n sub-paths $p_{1},\ldots ,p_{n}$. They are assigned to n MSNs, respectively. The generated path ring p starts and ends at the same location, forming a ring route. Thus, the USVs take different sub-paths by moving along the path ring clockwise or counter-clockwise in different sensing cycles. In this scheme, each SLoI can be visited and sensed within the objective time interval $t_{bdt}$. 

The proposed Hexagonal Grid-based Survey Planner (HGSP) for multiple MSNs is summarized in Algorithm 2 with pseudo code.

**Algorithm 2:** hexagonalGridBasedSurveyPlanner**Input**: $\hat{A},e_{bdt},t_{bdt},t_{M},v,sp_{1},r_{0},k$. **Output**: $n,P=\left(p_{1},\ldots ,p_{n}\right)$. 1   $r\prime =r_{0}$; 2   **do** 3     $r=r^{\prime }$; r′=r′−k; 4     $\left[num,S\right]=samplingframe\left(\hat{A},r\right)$; 5   **while** ($num\cdot \sqrt{3}r\prime \leq e_{bdt}$);  % Find the objective r 6   $V_{T}=MSTVertexCreation\left(S\right)$; 7   $E_{T}=MSTConstruction\left(V_{T},\prime {\mathrm{Kruskal}}^{\text{'}}\right)$;  % Construct the MST 8   $p=clockwiseCircumnavigationPathGeneration\left(S,V_{T},E_{T},sp_{1}\right)$; 9   p=circumnavigationPathUpdate(p);  % Generate the path ring 10  $t_{p}=pathTimeConsumption\left(p,v,t_{M}\right)$; 11  **if**
$t_{p}\leq t_{bdt}$
**then**
n=1; P=p; 12  **else**
$\left[n,P\right]=subPathDivision\left(p,v,t_{M},t_{bdt}\right)$;  % Generate the sub-paths for MSNs

## 4. Online Water Quality Index

Most WQIs (including the CCME WQI) focus on off-line evaluations, which utilize data collected at a low sampling rate (typically at monthly or quarterly intervals) and at a limited number of sampling locations. The CCME WQI has been widely used in water quality monitoring programs. The index formulation of the CCME WQI incorporates three statistical factors by comparing the measurements of water quality parameters and their guidelines (a range of acceptable values). The index formulation is based on the following three assessment factors [33]:

- **Scope** assesses the percentage of water quality parameters that do not meet their guidelines over the time period of interest:
  (2) $$F_{1}(\mathrm{Scope})\;=\frac{Number\;of\;failed\;parameters}{Total\;number\;of\;parameters}\times 100.$$
- **Frequency** represents the percentage of individual tests that failed the acceptable ranges over the time period of interest:
  (3) $$F_{2}(\mathrm{Frequency})\;=\frac{Number\;of\;failed\;tests}{Total\;number\;of\;tests}\times 100.$$
- **Amplitude** measures the degree by which the failed tests deviated from the acceptable levels. It is calculated in three steps as follows:
  (i) The fractional deviation of a failed test value from its acceptable limit is termed an “*excursion.*” When the failed test value is greater than the acceptable upper limit of its relative guideline:
    (4) $$excursion_{i}=\frac{Failed\;test\;value_{i}}{Guideline_{i}}-1.$$
    When the failed test value is less than the allowable lower limit of its relative guideline:
    (5) $$excursion_{i}=\frac{Guideline_{i}}{Failed\;test\;value_{i}}-1.$$
  (ii) The Normalized Sum of Excursions (*NSE*) is defined as the average value of excursions:
    (6) $$NSE=\frac{\sum_{i=1}^{n}excursion_{i}}{Total\;number\;of\;tests}.$$
  (iii) Amplitude Factor scales the *nse* to a value in the range 0-100 by an asymptotic function:
    (7) $$F_{3}(\mathrm{Amplitude})\;=\frac{NSE}{NSE+1}\times 100.$$
- The final index, ranging from 0 to 100 with a higher score representing a better quality, is calculated as the root mean square of these three factors as follows:
  (8) $$CCME\;WQI=100-\sqrt{\frac{F_{1}^{2}+F_{2}^{2}+F_{3}^{2}}{3}}.$$

In this section, the original index formulation of the CCME WQI is first expressed in an analytical form. Using that, it may be applied (with possible modification) to automated online data measurement at a relative high sampling rate (at minute or hour intervals). Furthermore, the derivation of the analytical form facilitates its further implementation on the IoT platform for online water quality indexing.

In the data acquisition process of the present work, the data records are measured and logged one by one. Each data record contains the sampling locations, sampling time, and the measurements of multiple water quality parameters. Each measurement is called a data sample or a test. The measurements involved in the indexing are expressed as:

(9) $$X={\left[\begin{array}{cc} \begin{array}{cc} x_{11} & x_{12}\\ x_{21} & x_{22} \end{array} & \begin{array}{cc} \ldots & x_{1l}\\ \ldots & x_{2l} \end{array}\\ \begin{array}{cc} \vdots & \vdots \\ x_{f1} & x_{f2} \end{array} & \begin{array}{cc} \vdots & \vdots \\ \ldots & x_{fl} \end{array} \end{array}\right]}_{f\times l},$$

where a row vector represents the measurements of l parameters of a data record, and f represents the number of samples involved in the index calculation. A guideline matrix is defined as

(10) $$G={\left[\begin{array}{cc} \begin{array}{cc} g_{11} & g_{12} \end{array} & \begin{array}{cc} \cdots & g_{1l} \end{array}\\ \begin{array}{cc} g_{21} & g_{22} \end{array} & \begin{array}{cc} \cdots & g_{2l} \end{array} \end{array}\right]}_{2\times l},$$

where $g_{1\cdot }$ and $g_{2\cdot }$ respectively represent the lower and upper acceptable values of the l parameters, according to their guidelines. For example, pH value, a common water quality parameter, is specified as $g_{1\mathrm{pH}}$ = 6.5 and $g_{2\mathrm{pH}}$ = 9 for the protection of aquatic life in freshwater, according to the Canadian Environmental Quality Guidelines (CEQG) [34].

To describe whether a data sample in X has failed compared to its guideline, a Boolean variable $b_{ij}$ corresponding to a function $B\left(x_{ij}\right)$, i = 1, …, f, j = 1, …, l, is defined as

(11) $$b_{ij}=B\left(x_{ij}\right)=\{\begin{array}{cc} 0 & x_{ij}\in \left[g_{1j},g_{2j}\right]\\ 1 & x_{ij}\notin \left[g_{1j},g_{2j}\right] \end{array}.$$

According to this formulation, the number of failed parameters is given by $p=\sum_{j=1}^{l}\underset{1\leq i\leq f}{\min}\left(b_{ij}\right)$; and the number of failed tests is given by $q=\sum_{i=1}^{f}\sum_{j=1}^{l}b_{ij}$. Then, the three assessment factors in the CCME WQI can be analytically expressed as follows:

$$F_{1}(\mathrm{Scope})\;=\frac{p}{l}\times 100$$

$$F_{2}(\mathrm{Frequency})\;=\frac{q}{f\times l}\times 100$$

$$excursion_{ij}=\{\begin{array}{cc} \left(\frac{g_{1j}}{x_{ij}}\right)-1 & x_{ij}<g_{1j}\\ 0 & g_{1j}\leq x_{ij}\leq g_{2j}\\ \left(\frac{x_{ij}}{g_{2j}}\right)-1 & x_{ij}>g_{2j} \end{array}$$

(12) $$F_{3}(\mathrm{Amplitude})\;=\frac{\sum_{i=1}^{f}\sum_{j=1}^{l}excursion_{ij}}{\sum_{i=1}^{f}\sum_{j=1}^{l}excursion_{ij}+f\times l}\times 100$$

The impact of a failed test, $x_{ij}\notin \left[g_{1j},g_{2j}\right]$ on the three factors will be different since a failed test causes different score changes in the three factors. In this work, the increment ΔF in a factor score caused by a failed test is defined as the factor sensitivity S. The sensitivities of the three assessment factors are given in Table 2.

According to the sensitivity expressions in Table 2, biased factor sensitivity may exist when it is applied to handle large amounts of online measurements (f≫l). On one hand, the Scope Factor may dominate the index score with only a few failed tests. For example, a failed test among the total of f×l tests produces the change $\Delta F_{1}=\frac{1}{l}\times 100$ in the first factor, which is f times larger than the change in the second factor $\Delta F_{2}=\frac{1}{f\times l}\times 100$, for the same cause. On the other hand, the parameter with a wide data range may lead to a score bias in the third factor, according to the expressions of the terms $excursion_{ij}$ and $S_{3}$. For example, failed electrical conductivity tests (data ranging from 0 to over $10^{4}\;\mu S/\mathrm{cm}$) may easily dominate the Amplitude Factor compared to failed pH value tests (data ranging from 0 to 14) in a large number of data records.

To avoid the score bias in the Scope Factor, the average of all measurements of a parameter, ${\bar{x}}_{\cdot j}$, is used for comparison with its guideline. Specifically, the Scope Factor is modified as follows:

(13) $$F_{1}^{\prime }(\mathrm{Scope})\;=\frac{\sum_{j=1}^{l}B\left({\bar{x}}_{\cdot j}\right)}{l}\times 100.$$

To avoid the score bias in the Amplitude Factor as caused by the excursion of failed tests, the term is modified according to the following normalization process:

(14) $$exursion_{ij}^{\prime }=\{\begin{array}{cc} \frac{g_{1j}-x_{ij}}{g_{1j}-MIN_{j}} & x_{ij}<g_{1j}\\ 0 & g_{1j}\leq x_{ij}\leq g_{2j}\\ \frac{x_{ij}-g_{2j}}{MAX_{j}-g_{2j}} & x_{ij}>g_{2j} \end{array},$$

where MAX and MIN represent the maximum and minimum values, respectively, that a certain parameter can reach. In the original formulation, $excursion_{ij}\in \left[0,\;\frac{MAX_{j}}{g_{2j}}-1\right]$ or $\in \left[0,\;\frac{g_{1j}}{MIN_{j}}-1\right]$. In the modified formulation, $excursion_{ij}^{\prime }\in \left[0,\;1\right]$ for all parameters. Thus, parameters with different variation ranges will result in an unbiased index score within the modified Amplitude Factor. The modified Amplitude Factor is derived using the modified term $excursion_{ij}^{\prime }$ given by (14):

(15) $$F_{3}^{\prime }(\mathrm{Amplitude})\;=\frac{\sum_{i=1}^{f}\sum_{j=1}^{l}excursion_{ij}^{\prime }}{\sum_{i=1}^{f}\sum_{j=1}^{l}excursion_{ij}^{\prime }+l}\times 100.$$

Note that f×l term in the original formulation (12) is replaced by l in the modified formulation. The aim is to reduce the large weighting of this term, as introduced by a large volume of online measurements. The Frequency Factor remains in its original form, as it represents the frequency of failed tests with a reasonable factor of sensitivity. The sensitivities of the modified factors are given in Table 3. This modified index is the Online Water Quality Index (OLWQI):

(16) $$OLWQI=100-\sqrt{\frac{F_{1}^{\prime }{}^{2}+F_{2}^{\prime }{}^{2}+F_{3}^{\prime }{}^{2}}{3}}.$$

## 5. Hardware Description

### 5.1. Mobile Sensor Node

Various water parameters can be monitored through automated sensing [35]. These parameters include flow rate, temperature, air pressure, pH value, dissolved oxygen, electrical conductivity, oxidation-reduction potential, nitrogen, phosphate, organic matter, microorganisms, and so on. Selection of the water quality parameters is based on the specific end use and the monitoring objective. In the developed platform, five sensors are implemented in each MSN to measure five representative parameters. They are listed below.

1. **Temperature sensor (T)** senses water temperature through a thermoresistive probe whose resistance increases with the heat transferred from the aquatic source. Many parameters are affected by temperature. Thus, temperature compensation is required during the sensor calibration for those parameters.
2. **pH Value sensor (pH)** measures the output voltage of an electrode due to the hydrogen ion activity in the water, which can then be translated into the pH value according to the hydrogen ion concentration.
3. **Dissolved Oxygen (DO) sensor** measures the output voltage of the sensor with an anode and a cathode, which is proportional to the concentration of the dissolved oxygen in the water.
4. **Electrical Conductivity (EC) sensor** measures the resistance of a two-pole cell of the sensor. Water conductivity is proportional to the conductance (the inverse of the resistance) of the sensor.
5. **Oxidation-Reduction Potential (ORP) sensor** measures the output voltage between a measuring electrode and a reference electrode, which indicates the ability of a water body to acquire electrons, thereby to be reduced.

**Figure 4:**
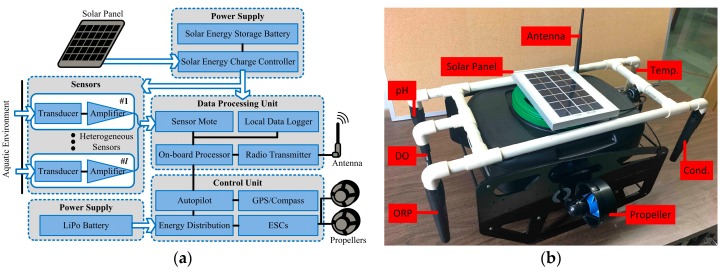
(**a**) The design framework of the Mobile Sensor Nodes (MSN); (**b**) The developed MSN in the IoT platform.

The designed framework and the developed MSN are shown in Figure 4. Each node consists of five sensors, a control unit, a data processing unit, and two power supply modules. To avoid the inaccurate measurements caused by surrounding objects (the magnetic field between the electrodes may be affected by the nearby surroundings), the five sensors are held separately through a PVC structure. All electronic components are deployed inside a waterproof floating buoy. The conversion of the sensor output signals (e.g., voltages) to the sensor readings, which indicate the real concentrations, is carried out by a mote, Waspmote with microcontroller ATmega1281, which is an advanced mote manufactured by Libelium Communicaciones Distribuidas S.L. Data is processed at the on-board processor, Raspberry Pi 3, and then transmitted to the BS through a Wi-Fi or Zigbee radio transmitter. To enable mobility, a BlueRobotics ROV external structure with two T200 propellers are integrated. A 3DR Pixhawk mini with a GPS module is equipped as the autopilot of the MSN. To avoid data missing due to package loss or communication failure during data transmission, recently collected data is stored at the local data logger. A 3.7 V, 6600 mA∙h ion polymer rechargeable battery is used to provide power for sensing and wireless transmission. It also stores the harvested energy from a 23,016,020 mm solar panel. In addition, a 14.8 V, 10 A∙h LiPo battery is used to supply power for the autopilot, the Electronic Speed Controllers (ESCs) and the propellers.

### 5.2. Base Station and Remote Server

**Figure 5:**
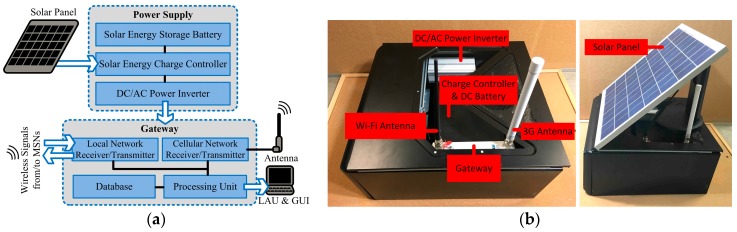
(**a**) The design framework of the Base Station (BS); (**b**) The developed BS in the IoT platform.

The design framework with the developed BS are shown in Figure 5. It consists of a gateway, an LAU, and a power supply module. The gateway, the Meshlium Xtreme manufactured by Libelium, is deployed in the BS. It is a Linux-based router, which works as the gateway for the local wireless network. The radio receiver of the gateway receives the data transmitted from the distributed MSNs. The data received from the MSNs is stored in an MySQL database embedded in the gateway. The quality indexing algorithm is operated at the LAU. The monitoring results can be accessed at the BS by a laptop (LAU) through a local Graphic User Interface (GUI), mainly for local examination by technicians in the field. The data is then transmitted to the RS as well. The gateway accesses the cellular network to communicate with the RS via the Internet. The solar energy is collected by a 48,043,030 mm solar panel and stored into a 12 V DC battery through a solar energy charge controller. Then 110 V AC power is supplied to the gateway through a DC/AC power inverter, which is connected to the charge controller. 

The RS is a PC with a 4.00 GHz Intel Core i7-6700K CPU and a 32 GB RAM. By implementing the proposed survey planner, the sensing missions including the sampling locations and the paths for MSNs are generated by a CAU at the RS. The missions are then transmitted to the MSNs via the BS. The RS also receives the data collected in the field and interprets the data online for users.

## 6. Experimental Results and Discussion

**Figure 6:**
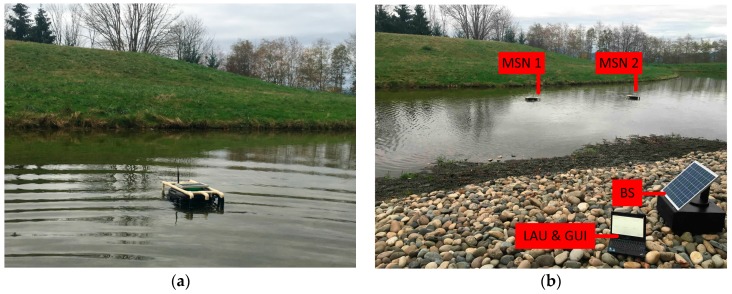
Field deployment of the developed platform at the Yosef Wosk Reflecting Pool: (**a**) Deployment of the MSN in the pool; (**b**) Deployment of the platform in the field.

The developed rapidly deployable IoT platform has been deployed at the Yosef Wosk Reflecting Pool of The University of British Columbia, Canada. The in situ deployment of the platform is shown in Figure 6.

A fully charged battery enables an MSN to move continuously around 80 min at the average speed v of 0.4 m/s. Thus, the total travel distance of a fully charged MSN is approximately 1920 m. In the experiment, 20% battery capacity, i.e., 4 A∙h, was assigned for each sampling cycle. Thus, the energy budget for a sampling cycle was $e_{bdt}$ = 384 m. The time interval $t_{bdt}$ was set to 15 min. The time cost $t_{M}$ for measuring process at each SLoI was set to 10 s. Given the above configurations and the contour of the study area, the proposed Hexagonal Grid-based Survey Planner was operated at the RS to generate the survey missions for MSNs in the field. The remaining initial inputs were set to: $r_{0}$ = 3 m and k = 0.2 m. Then the results were obtained by r = 2.4 m, d = 4.2 m, num = 88, |SP| = 88, |p| = 366 m $\leq e_{bdt}$, n=2, where 88 sampling locations were covered by the generated path. Accordingly, two MSNs were required in the field to carry out the survey mission to meet the time interval requirement $t_{bdt}$ = 15 min. The theoretical time interval for data collection at each sampling location can be estimated as $t_{p}=t_{M}\cdot \lceil num/n\rceil +d\cdot \lceil num/n\rceil /v=897$ s $<t_{bdt}$.

**Figure 7:**
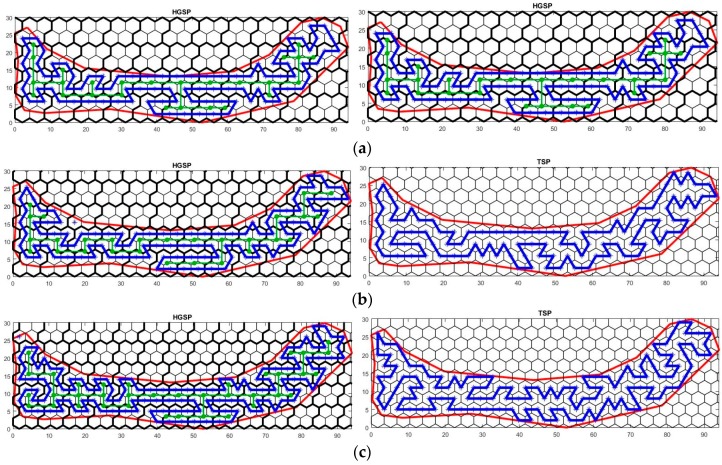
Generated survey plan using Hexagonal Grid-based Survey Planner (HGSP) and Travelling Salesman Problem (TSP) with respect to different cell sizes: (**a**) r=2.4 m, d=4.2 m; (**b**) r=2.2 m, d=3.8 m; (**c**) r=2.0 m, d=3.5 m.

Note that without generating the final path, the objective cell size r can be obtained by referring to the total number of SLoIs after cellular decomposition (line 1–5 in Algorithm 2), such that the total path length is less than the energy budget $e_{bdt}$. In order to evaluate the performance of the proposed HGSP algorithm for generating the circumnavigation path (line 6–9 in Algorithm 2), it was operated with respect to different cell sizes in comparison with the TSP approach solved by linear integer programming. The experiments were executed using Matlab R2017a in the PC with a 4.00 GHz Intel Core i7-6700K CPU, 32 GB RAM and the experimental results are given in Table 4. Due to the limited space, some of the plan views by applying the HGSP and TSP algorithms are shown in Figure 7.

The solution of the TSP approach leads to the minimum path length for traversing all points of interest, which provides optimal performance on energy and time efficiency. In Table 4, both the proposed HGSP algorithm and the TSP algorithm guaranteed the optimal path length when all SLoIs were visited. However, the performance on the algorithm time cost was decreased by using the proposed approach. For some cases where unvisited SLoIs existed, the total path length was still optimal for covering all other SLoIs. The low quantity of unvisited SLoIs was because they had only one neighboring SLoI, and hence no path segments could be generated for them (see top left unvisited SLoI in Figure 7c).

**Figure 8:**
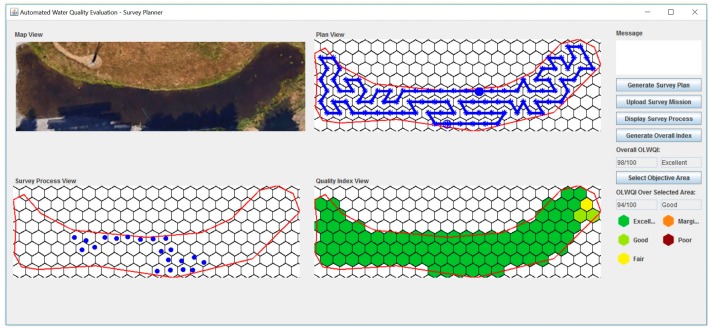
Graphic User Interface (GUI).

The platform core software was developed in Java and executed on the RS to implement the proposed HGSP algorithm and the OLWQI algorithm. The geometric map of the study area was embedded in the software. The survey mission was generated at the core and then sent to MSNs via the BS. The Robotic Operating System (ROS) was executed on the Raspberry Pi 3 in the MSN to control and navigate the MSN to move to the objective sampling locations that included in the survey mission. The collected data was transmitted to the RS via the BS and stored in the MySQL database. The core software then processed the collected data to calculate the OLWQI. Before the MSNs were deployed in the water body, all sensors in each node were calibrated carefully in the field to assure good data sample quality in the initial stages. In addition, two MSNs were fully charged and launched at the predetermined location. In the field test, the coordinates of the SLoIs were translated from the meter scale to GPS coordinates at the RS, and uploaded as the waypoints to the MSNs via the BS. The two MSNs passed along the target path ring four times, while each sampling location was measured eight times over two hours. Figure 8 shows a GUI display during the experiment. The path was planned based on the energy budget and the sampling frequency requirement that were set using the “Generate Survey Plan” function in the GUI. The resulting planned path was displayed in the “Plan View”. The blue circles in the plan view denote the division points for the sub-paths, where the hollow one indicates the initial launching position of the MSNs. After successfully uploading the survey mission to the MSNs, the in situ data sampling locations of the MSNs could be checked during the survey process through the “Survey Process View” in the GUI.

The proposed OLWQI is primarily intended for short-term water quality evaluation using data collected through automated sampling. The category interpretation based on the index score referred to Excellent: 95–100; Good: 80–94; Fair: 65–79; Marginal: 45–64; Poor: 0–44 [33]. The algorithm for calculating the OLWQI was implemented in the LAU and CAU, where the temporal and spatial scales could be set in a flexible manner by user inputs. In the experiments, a one-hour window was used to calculate the OLWQI (four sampling cycles are involved, inv=4) in a temporal point of view. Meanwhile, the quality index of each cell, the overall study area, and a selected objective area were provided and displayed in the GUI (see Figure 8). The quality indexing results at all the fine hexagonal cells were displayed in the “Quality Index View”, indicated by five different colors referring to the five quality categories. The overall OLWQI were calculated by the integration of the data collected at all the SLoIs over the study area. By utilizing the “Select Objective Area” function in the GUI, the users could check the indexing results across any objective area that was selected in the view.

**Figure 9:**
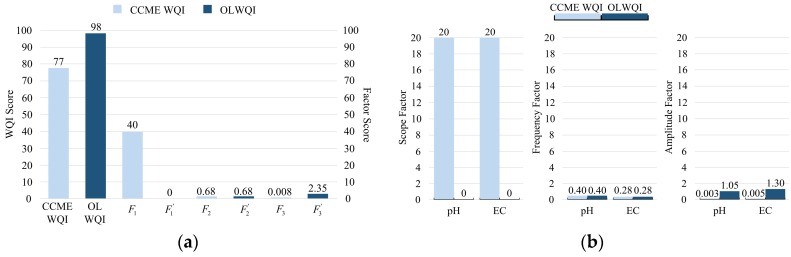
Comparison of the CCME WQI and the OLWQI: (**a**) Quality indexing results; (**b**) Factor scores due to the failed tests.

To demonstrate the performance of the OLWQI in comparison with the CCME WQI, both indices have been implemented in the experiment. In Figure 9, data samples were selected from a one-hour time window to illustrate the indexing results of the overall study area (overall WQI) by using these two indices. In the time window, seven tests of pH value and five tests of EC have failed (readings do not meet their relative guidelines, pH: 6.5–9, EC: <2000 µS/cm) among the total 1760 tests (f·l=num·inv·l=88 × 4 × 5 = 1760). This means 0.68% tests failed in all tests. As shown in Figure 9a, the overall index score of the CCME WQI was 77/100 (Fair). The Scope Factor dominated the total index score of the CCME WQI. Particularly, in Figure 9b, the Scope Factor in the CCME WQI was degraded by 40 units due to these few failed tests, leading to the unreliable index score of the CCME WQI. In fact, in the worst-case scenario, only two failed tests from these two parameters (pH value and EC) can cause such a 40-unit degradation. By contrast, the OLWQI indicated an online indexing result of 98/100 (Excellent). The Scope Factor in the OLWQI has not been affected unreasonably by the small number of failed tests.

The influence of the Amplitude Factor can be ignored if the CCME WQI is implemented on a large volume of online sampled data. This issue is demonstrated in Figure 9. The modification in the OLWQI for the Amplitude Factor makes it effective for a large volume of data. In addition, the water quality parameters with different data ranges result in different excursion ranges for the Amplitude Factor when the CCME WQI is implemented. Notice that the parameter with a large value range (e.g., EC) results in a large excursion range. This may lead to a biased index score. For example, a test of EC = 3200 μS/cm leads to the excursion of 0.6 in the Amplitude Factor of the CCME WQI. It even exceeds the upper limit of the pH excursion, since the maximum pH value is 14 (maximum pH excursion: 14/9−1=0.56). Hence, a parameter may dominate the Amplitude Factor in the CCME WQI. In contrast, the OLWQI provided the same excursion range (from 0 to 1) for different parameters, regardless of their data range and guidelines.

In the above experiment, data samples collected from the field test were used to demonstrate the performance results of the OLWQI compared to the state-of-the-art CCME WQI. For further validation of the performance of online indexing, both indices have been implemented on the realistic dataset with a large volume of continuous online measurements collected by the Chesapeake Bay Interpretive Buoy System (CBIBS) [4]. The data from 1 June 2017 to 1 July 2017 at 10 monitoring stations in the dataset have been selected for the experiment. The measurements of six parameters that are related to water quality (i.e., Chlorophyll A, Dissolved Oxygen, Turbidity, Conductivity, Salinity, and Temperature) have been involved in the index calculation. The index were calculated hourly by aggregating the latest 24-h data for quality evaluation (24-h sliding time window). Due to the limited space, Table 5 shows some of the indexing results when implemented the CCME WQI and the proposed OLWQI on the dataset. 

To demonstrate the resulting factor effects on the final index score, we have statistically summarized both indices by applying stepwise regression analysis. Two examples have been given in Table 6 to show the statistical summary of the final index versus the three factors utilizing the one-month data from the single station (First Landing Station), and the data from the regional multiple stations (First Landing Station and York Spit Station). Both examples in the table demonstrated that the Scope Factor in the CCME WQI dominated the whole index by referring to the adjusted R-squared value of step 1. Meanwhile, the Amplitude Factor in the CCME WQI had very limited influence on the final index score by referring to the adjusted R-squared value of step 3. In the experiment, the stepwise regression analysis has been executed 20 times on the selected dataset, including 10 times on the 10 single stations, and 20 times on the pairs of neighboring stations. The statistical results are summarized in Table 7. In the table, $f_{F_{i}}=\sum_{j=1}^{n}R_{ij}/N$, where n = the number of times that the factor $F_{i}$ was obtained in the first step, i=1,…,3; $R_{ij}$ = the corresponding adjusted R-squared value in the first step of the factor $F_{i}$; N = the number of the total experimental runs. The experimental results demonstrated that the proposed OLWQI provided the indexing results with a more balanced factor sensitivity for large quantities of online data automatically collected, compared to the CCME WQI.

## 7. Conclusions

This paper presented a hexagonal grid-based survey planner and an online quality index that were implemented on an IoT platform. The developed platform provided a cost-effective, fast, deployable, and easily maintainable solution for the high-resolution spatiotemporal telemoitoring of surface water. In the developed platform, the proposed survey planner generated a shortest circular path to cover the sampling locations of interest that are uniformly distributed over the study area, under the energy constraints of the mobile sensor nodes. Multiple mobile sensor nodes were positioned uniformly to travel along the planned path such that each objective sampling location could be visited within the time interval requirement. The proposed sensor scheduling and path planning algorithm was designed for the application scenario of automated water quality monitoring. This automation would be calculated according to the limited power supply of each mobile sensor and required sampling frequency, determining the more evenly distributed sampling locations that the mobile sensors can measure to gather more information and explore the unknown field. In addition, an online quality index, the OLWQI, was modified based on the state-of-the-art water quality index, CCME WQI, to evaluate water quality by integrating a large volume of online data acquired through automated sampling. Meanwhile, the OLWQI that was formulated in the analytical form facilitated online processing by automatic execution on the automated devices.

The developed IoT platform has been deployed in a real water source to demonstrate the implementation of the overall system. The experimental results on path planning demonstrated the effective and efficient performance of the proposed survey planer compared to the state-of-the-art TSP algorithm for generating the coverage path. The performance of the OLWQI in compassion to the existing CCME WQI was demonstrated by using the realistic data that was collected through our developed platform and the data from the CBIBS dataset. The experimental results show that the proposed OLWQI provided balanced factor sensitivities and reliable evaluation results for online quality indexing compared to the CCME WQI. The OLWQI, expressed in the analytical form, facilitated online processing by automatic execution on the devices. In future work, the proposed platform will be extended to exploit the information collected from the mobile sensor nodes to modify the objective sampling locations adaptively over time by incorporating environmental model adaptation. The proposed approach in this paper can be utilized as an initial deployment to collect prior knowledge of the potential environmental model.

## Figures and Tables

**Table 1:** MST Vertex Creation.

Coarse Cell	Condition & Strategy
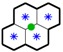	SLoIs exist in 1, 2, 3, 4 fine-cells. A vertex is created at the midpoint of the edge between 2 and 3 fine-cells.
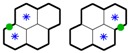	Left (Right) figure: SLoIs exist in 1, 3 (2, 4) fine-cells. A vertex is created at the left (right) side of the coarse cell if its left (right) neighboring coarse cell has four SLoIs.
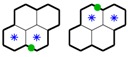	Left (Right) figure: SLoIs exist in 1, 2 (3, 4) fine-cells. A vertex is created at the bottom (top) side of the coarse cell if its bottom (top) neighboring coarse cell has four SLoIs.
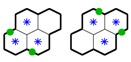	Left (Right) figure: SLoIs exist in 1, 2, 3 (2, 3, 4) fine-cells. A vertex is created with the same strategies by considering this coarse cell as a 1, 2 and 1, 3 (2, 4 and 3, 4) coarse cell. If two vertices are created, select a random one and remove the other one.
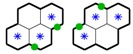	Left (Right) figure: SLoIs exist in 1, 2, 4 (1, 3, 4) fine-cells. A vertex is created with the same strategies by considering this coarse cell as a 1, 2 and 2, 4 (1, 3 and 3, 4) coarse cell. If two vertices are created, select a random one and remove the other.

**Table 2:** Factor sensitivity of the Canadian Council of Ministers of Environment (CCME) Water Quality Index (WQI).

Factor	Factor Sensitivity
Scope	$S_{1}=\frac{1}{l}\times 100$
Frequency	$S_{2}=\frac{1}{f\times l}\times 100$
Amplitude ^1^	$S_{3}=\frac{f\times l}{\sum^{}excursion+f\times l+\frac{{\left(\sum^{}excursion+f\times l\right)}^{2}}{excursion_{ij}}}\times 100$

^1^ The derivation of the sensitivity is given in the appendix.

**Table 3:** Factor sensitivity of the Online Water Quality Index (OLWQI).

Factor	Factor Sensitivity
Scope	$S_{1}^{\prime }=\frac{1}{f\times l}\times 100$
Frequency	$S_{2}^{\prime }=\frac{1}{f\times l}\times 100$
Amplitude	$S_{3}^{\prime }=\frac{l}{\sum^{}excursion^{\prime }+l+\frac{{\left(\sum^{}excursion^{\prime }+l\right)}^{2}}{excursion_{ij}^{\prime }}}\times 100$ $excursion_{ij}^{\prime }\in \left[0,\;1\right]$

**Table 4:** Algorithm Performance.

*r*	*d*	*num*	Visited SloIs |SP|		Total Length |p|		Algo. Time Cost	
			HGSP	TSP	HGSP	TSP	HGSP	TSP
2.6 m	4.5 m	73	72	73	324 m	332 m	0.53 s	2.64 s
2.4 m	4.2 m	88	88	88	366 m	366 m	0.60 s	3.17 s
2.2 m	3.8 m	101	100	101	381 m	385 m	0.72 s	26.24 s
2.0 m	3.5 m	127	126	127	436 m	442 m	0.93 s	335.57 s
1.8 m	3.1 m	159	159	159	496 m	496 m	1.31 s	178.1 s
1.6 m	2.8 m	203	203	203	563 m	N/A	1.79 s	N/A

N/A: Computation did not complete within the limit of 1000 s. The time cost is the average time consumption of 10 executions.

**Table 5:** Indexing Results using Data Collected at the First Landing Station in CBIBS.

Date & Time	$F_{1}$	$F_{2}$	$F_{3}$	CCME WQI	$F_{1}^{\prime }$	$F_{2}^{\prime }$	$F_{3}^{\prime }$	OLWQI
6/20/2017 13:00	16.67	6.94	0.92	90	0	6.94	7.46	94
6/20/2017 14:00	16.67	6.94	0.92	90	0	6.94	7.46	94
6/20/2017 15:00	16.67	6.94	0.92	90	0	6.94	7.46	94
6/20/2017 16:00	16.67	7.64	0.99	89	0	7.64	8.01	94
6/20/2017 17:00	16.67	8.33	1.20	89	16.67	8.33	9.59	88
6/20/2017 18:00	16.67	9.03	1.50	89	16.67	9.03	11.75	87
6/20/2017 19:00	16.67	9.03	1.52	89	16.67	9.03	11.89	87
6/20/2017 20:00	16.67	9.03	1.52	89	16.67	9.03	11.89	87
6/20/2017 21:00	16.67	9.03	1.52	89	16.67	9.03	11.86	87
6/20/2017 22:00	16.67	9.03	1.53	89	16.67	9.03	11.95	87

**Table 6:** Stepwise Regression Analysis: Final Index versus Three Factors.

	CCME WQI				OLWQI			
Single Station	Candidate Term	Step 1 (Coef)	Step 2 (Coef)	Step 3 (Coef)	Candidate	Step 1 (Coef)	Step 2 (Coef)	Step 3 (Coef)
	$F_{1}$	−0.5808	−0.5742	−0.5744	$F_{1}^{\prime }$	−0.8511	−0.2145	−0.2378
	$F_{2}$		−0.0690	−0.0611	$F_{2}^{\prime }$		−0.9327	−0.4381
	$F_{3}$			−0.0520	$F_{3}^{\prime }$			−0.3588
	$R^{2}\left(\mathrm{adj}\right)$	98.00%	99.91%	99.92%	$R^{2}\left(\mathrm{adj}\right)$	83.97%	98.75%	99.81%
Multiple Stations	Candidate Term	Step 1 (Coef)	Step 2 (Coef)	Step 3 (Coef)	Candidate	Step 1 (Coef)	Step 2 (Coef)	Step 3 (Coef)
	$F_{1}$	−0.5966	−0.5502	−0.5528	$F_{1}^{\prime }$	−0.8347	−0.3192	−0.3002
	$F_{2}$		−0.1821	−0.1536	$F_{2}^{\prime }$		−1.4212	−0.3049
	$F_{3}$			−0.0690	$F_{3}^{\prime }$			−0.4066
	$R^{2}\left(\mathrm{adj}\right)$	98.47%	99.94%	99.95%	$R^{2}\left(\mathrm{adj}\right)$	85.43%	97.95%	99.89%

**Table 7:** Statistical Results of the Factor Effects on the Final Index.

CCME WQI			OLWQI		
$f_{F_{1}}$	$f_{F_{2}}$	$f_{F_{3}}$	$f_{F_{1}^{\prime }}$	$f_{F_{2}^{\prime }}$	$f_{F_{3}^{\prime }}$
79.89%	3.64%	7.57%	15.75%	36.98%	37.88%

## Appendix A

Consider a failed test $x_{ij}\notin \left[g_{1j},g_{2j}\right]$, and denote the sum of excursions of the other tests by

$$\sum^{}excursion=\sum_{I=1}^{f}\sum_{J=1}^{l}excursion_{ij},\;I\neq i,\;J\neq i.$$

The sensitivity of the Amplitude Factor is derived as follows:

$$S_{3}=\frac{\sum^{}exursion+excursion_{ij}}{\sum^{}exursion+excursion_{ij}+f\times l}\times 100-\frac{\sum^{}exursion}{\sum^{}exursion+f\times l}\times 100\;=\frac{excursion_{ij}\times f\times l}{\left(\sum^{}exursion+excursion_{ij}+f\times l\right)\left(\sum^{}exursion+f\times l\right)}\times 100\;=\frac{excursion_{ij}\times f\times l}{\left(\sum^{}exursion+f\times l\right)\times excursion_{ij}+{\left(\sum^{}exursion+f\times l\right)}^{2}}\times 100\;=\frac{f\times l}{\left(\sum^{}exursion+f\times l\right)+\frac{{\left(\sum^{}exursion+f\times l\right)}^{2}}{excursion_{ij}}}\times 100.$$
